# Synchronous hepatocellular carcinoma and gallbladder adenocarcinoma with neuroendocrine differentiation: a case report and literature review

**DOI:** 10.1186/s12893-020-00905-6

**Published:** 2020-10-20

**Authors:** Yan Xu, Quan-Ning Chen, Hui Wang, Nan-Bin Liu, Bao-Min Shi

**Affiliations:** Department of General Surgery, Tongji Hospital, Tongji University Medical School, Shanghai, 200065 People’s Republic of China

**Keywords:** Hepatocellular carcinoma, Gallbladder cancer, Synchronous double primary cancer, Case report

## Abstract

**Background:**

Double primary cancers have a low incidence rate, and synchronous hepatocellular carcinoma and gallbladder adenocarcinoma are rarely reported. Here, we report such a case— the 12th case of synchronous double primary cancers featuring HCC and GC, but the first case of neuroendocrine differentiation in the gallbladder.

**Case presentation:**

A 77-year-old female was admitted to the hospital complaining of weakness and inappetence for six months. Contrast-enhanced computed tomography (CT) of the abdomen indicated an 11 cm space-occupying lesion in the right lobe of the liver. Later, magnetic resonance imaging showed a high possibility of a massive hepatoma, and multiple gallstones were also seen. After transhepatic arterial chemoembolization, a repeat abdominal CT showed obvious local nodular thickening in the gallbladder wall. Finally, resection of the right lobe of the liver and cholecystectomy were performed. During an approximately 2-year follow-up, the patient recovered uneventfully without recurrence or metastasis.

**Conclusion:**

The disease in this case is rare and lacked typical radiological features. More precise and advanced diagnostic techniques are needed to obtain a clear diagnosis and refine treatment strategies. The management strategy should always be curative, even in the presence of multiple malignancies.

## Background

Multiple primary cancers (MPCs) refer to two or more kinds of malignancies occurring in the same or different organs, with unknown pathogeneses. MPC involving both the liver and gallbladder is rarely reported, and only 15 cases have been recorded, with 11 cases histologically proven as hepatocellular carcinoma (HCC) and gallbladder adenocarcinoma (GC). Here, we present the 12th case of synchronous double primary cancers featuring HCC and GC, but the first case of neuroendocrine differentiation in the gallbladder.

## Case presentation

**Fig. 1:**
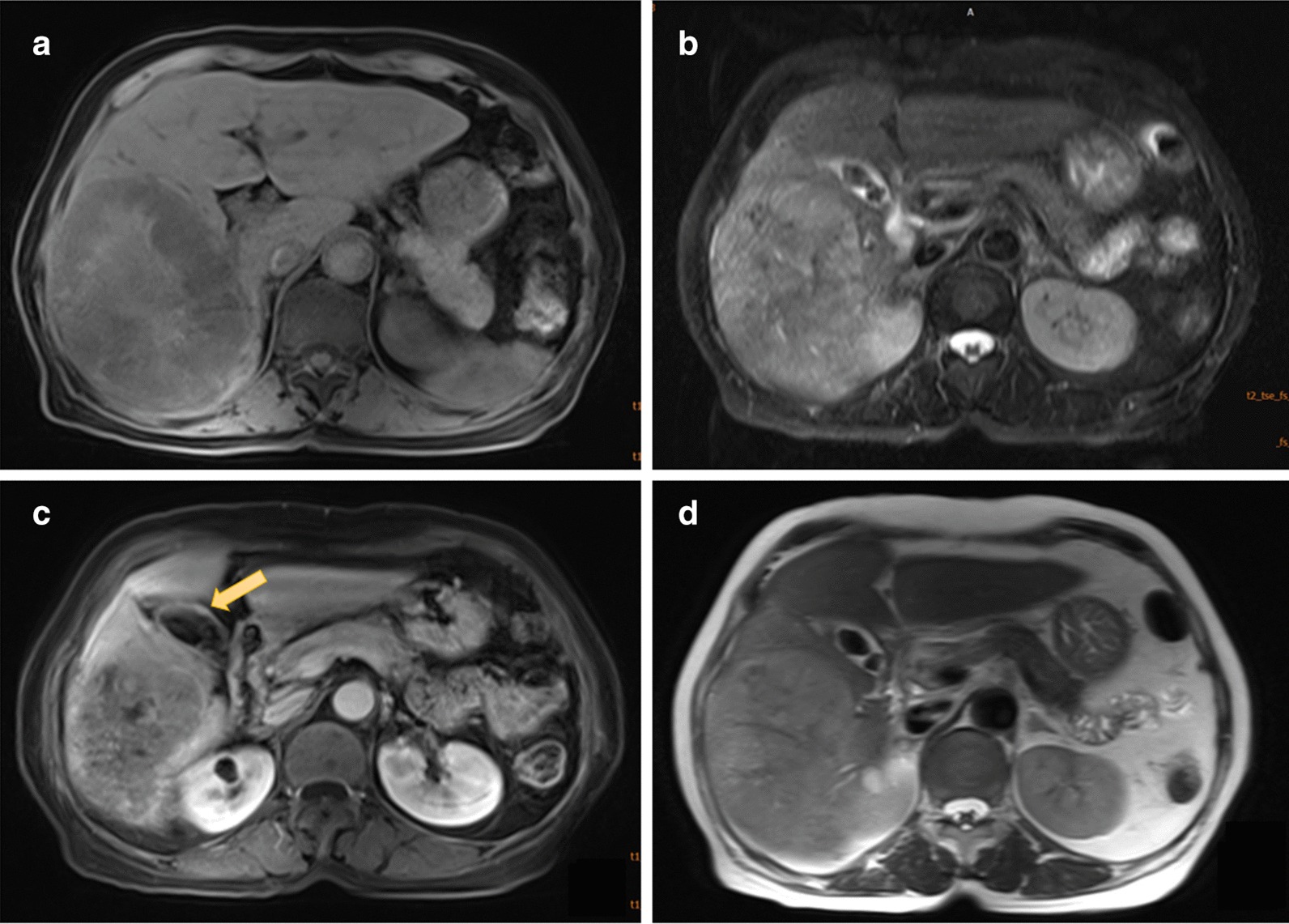
Abdominal MRI showed an 8.9 × 12.3 cm-sized mass, gallstones and slightly local nodular thickening in the gallbladder wall; **a** and **c** T1W images, **b** and **d** T2W images

In 2017, a 77-year-old retired female patient was admitted to our hospital, which serves as an outpatient hospital, with only complaints of weakness and inappetence for 6 months. A contrast-enhanced CT indicated an 11 cm space-occupying lesion in the right lobe of the liver. There was no particular family history or infectious medical history. She did not receive any medical intervention before admission, and her vital signs, such as blood pressure, heart rate, respiration rate and body temperature, were normal. Physical examination showed the appearance of mild cachexia, no significant abdominal symptoms such as tenderness, rebound tenderness or muscular tension were noticed, and the abdomen was soft. Palpation revealed a normal liver and spleen, and normal borborygmus existed. The blood routine was normal, other blood chemistries showed a serum albumin of 29.3 (40–55 g/L), ALT of 21 (< 15U/L), AST of 98 (13-35U/L), T-Bil of 17.7 (5.1–20.5 µmol/L), CEA of 3.02 (< 4.7 ng/mL), CA19-9 of 7.49 (< 39U/mL) and CA12-5 of 20.35 (< 35U/mL), but a unusually high level of AFP (more than 1210 ng/mL). Serological tests for hepatitis viruses B and C were negative, as were other infectious indications. Then, the patient underwent an MRI examination that revealed a suspicion of a massive hepatoma (8.9 × 12.3 × 9.9 cm) and multiple gallstones. In fact, there was slight local nodular thickening in the gallbladder wall that was easily ignored (Fig. 1).

**Fig. 2:**
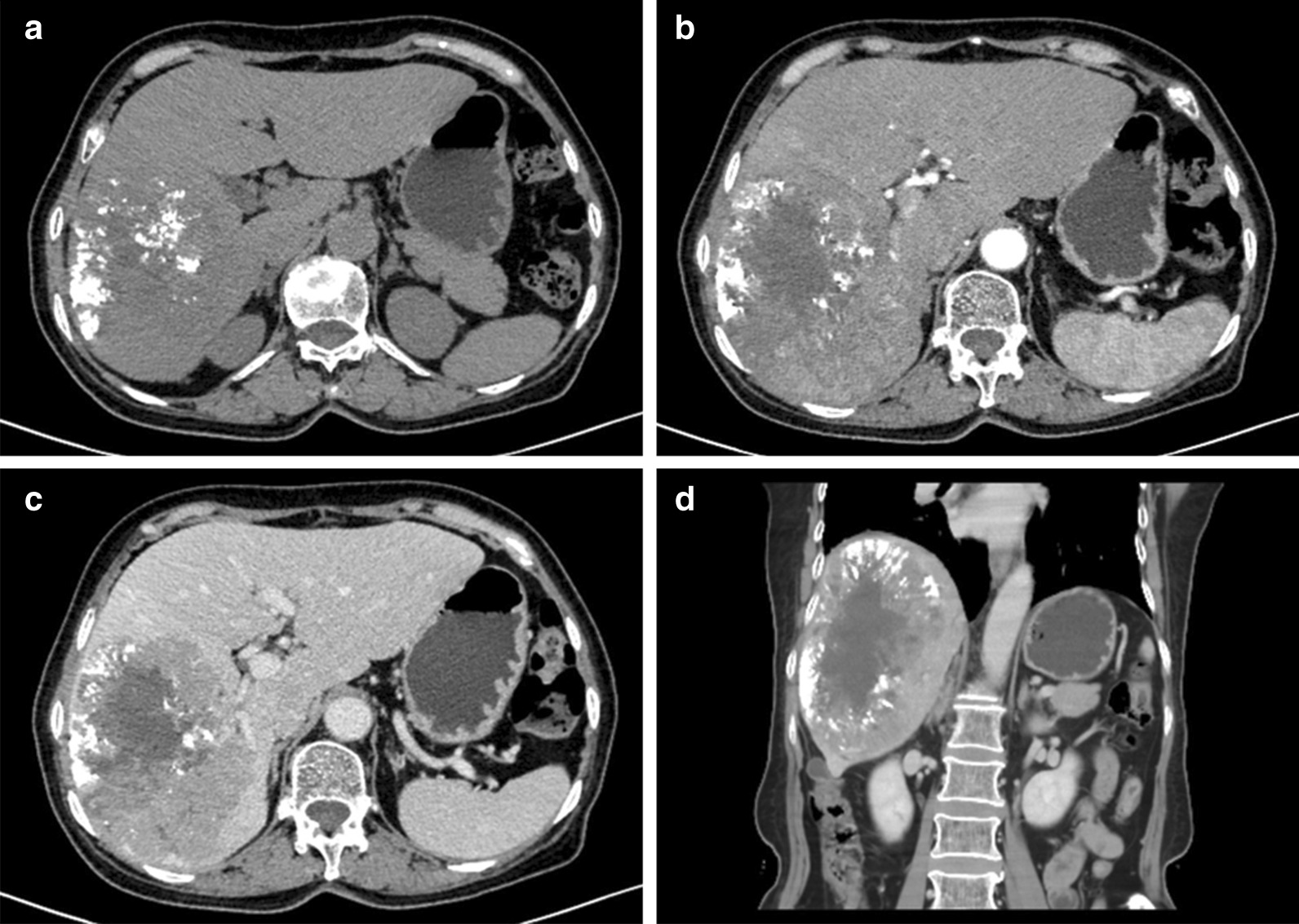
Abdominal CT images after TACE to observe the hepatoma. **a** Plain scan, **b** arterial phase, **c** venous phase, **d** coronal plane

**Fig. 3:**
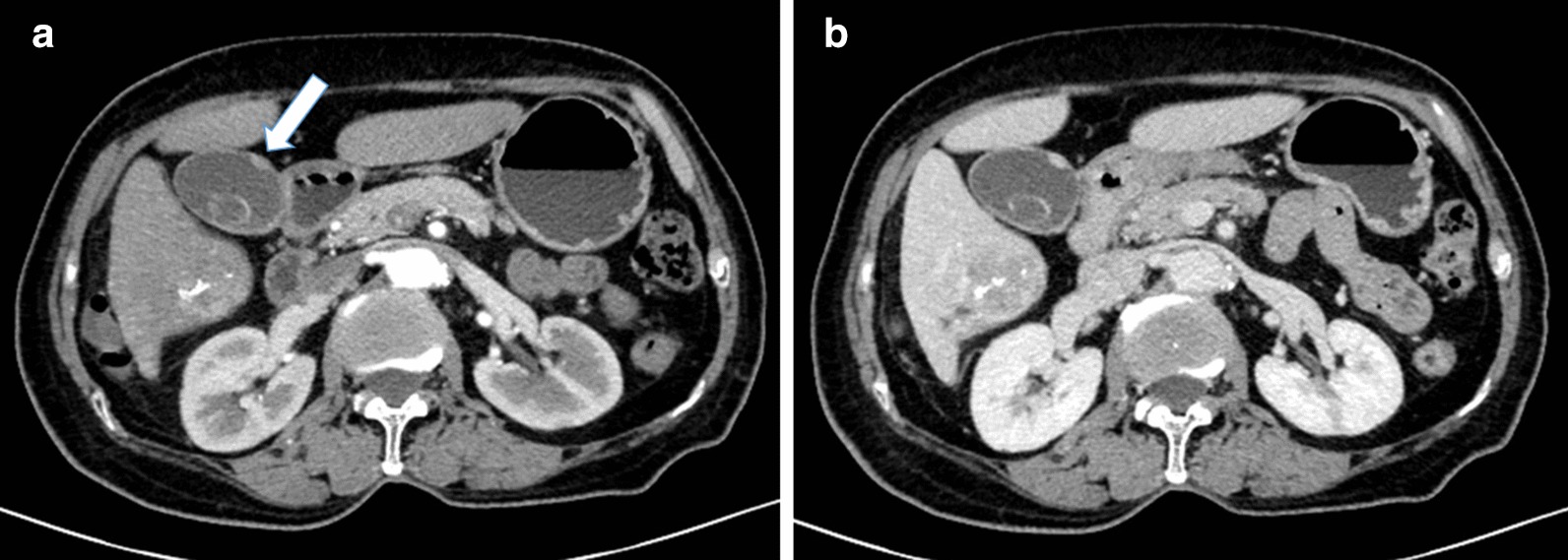
Abdominal CT images after TACE to observe the gallbladder mainly. **a** Arterial phase (white arrow indicating nodular thickening in the gallbladder wall), **b** venous phase

TACE is the most widely used primary treatment for unresectable HCC, and was the recommended first-line therapy for patient with intermediate-stage disease [1]. Liver resection remains the curative for various liver malignancies [2]. Currently the extent of liver resection/future liver remnants volume has been shown to most consistently and decisively determine the liver regeneration [3]. Truly some centers for radiation segmentectomy has also shown the encouraging results using selective internal radiation therapy(SIRT) as an alternative to curative therapies, but it is not supported by guidelines due to a lack of solid evidence. Therefore, considering the massive entity, the patient first underwent TACE to minimize the size of the mass. Approximately one and a half months later, a repeat abdominal enhanced CT was performed, showing the changes after TACE (Fig. 2). Additionally, the gallbladder lesion became more obvious (Fig. 3).

**Fig. 4:**
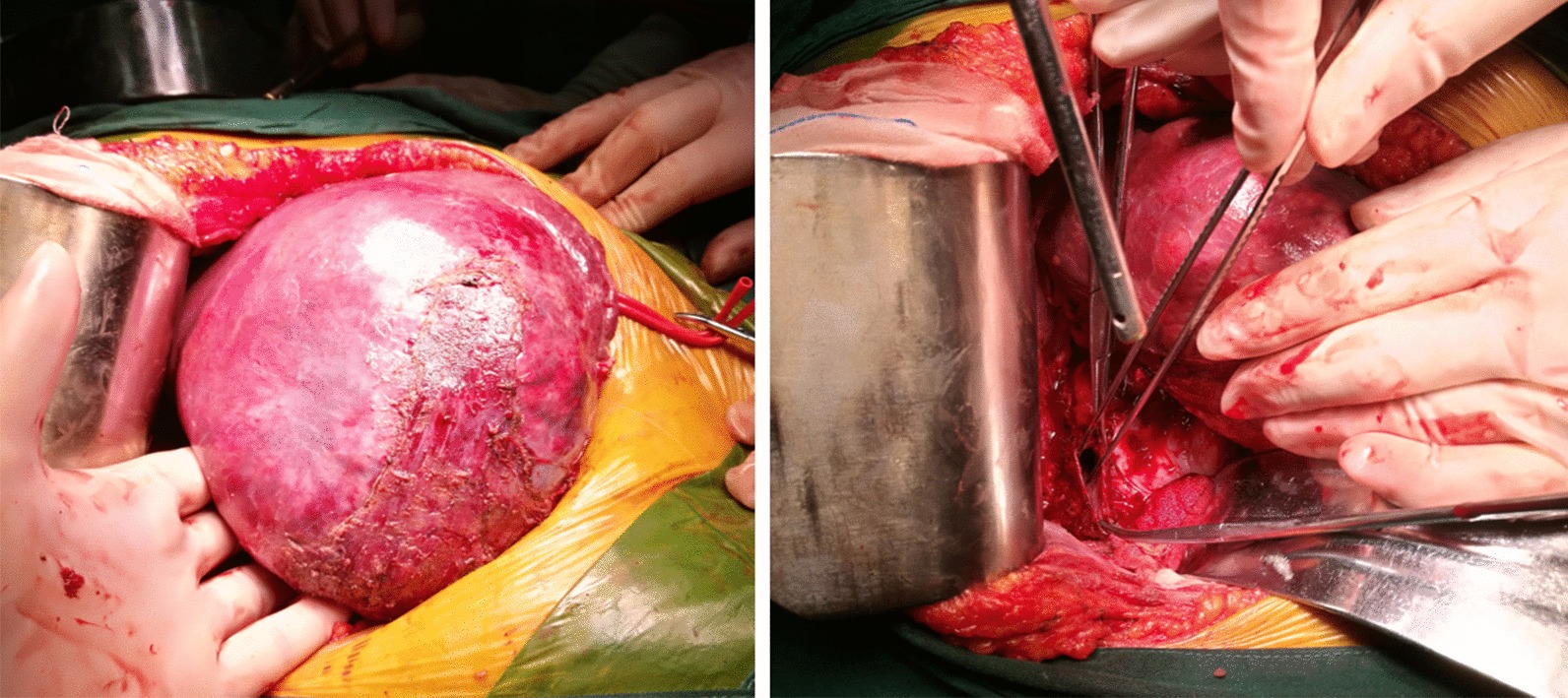
Intraoperative findings: the tumor located in the right lobe of the liver involved segments V, VI, VII, and VIII and partially invaded the right diaphragm

From the CT images it could be concluded that a future remnant liver volume of at least 30% of the original total liver volume, which met the current guidelines for extended liver resection [4]. Moreover, the tumor located in the right lobe of liver, without metastasis in either the peritoneal cavity or the rest of the liver. So routinely right hemi-hepatectomy and cholecystectomy were planned. After preoperative preparation and general anesthesia, the patient underwent surgery in the supine position performed by the chief physician, who has approximately 25 years of specialized training. Intraoperatively, the tumor located in the right lobe involved segments V, VI, VII, and VIII and partially invaded the right diaphragm (Fig. 4), and the size of the gallbladder was 6.5 × 3.5 × 2 cm, with the wall was partly thickened to 0.5 cm. Without adhesion and invasion, anatomy of hepatic portal area was clear, then complete resection of hepatoma with negative margin and lymphadenectomy were achieved.

**Fig. 5:**
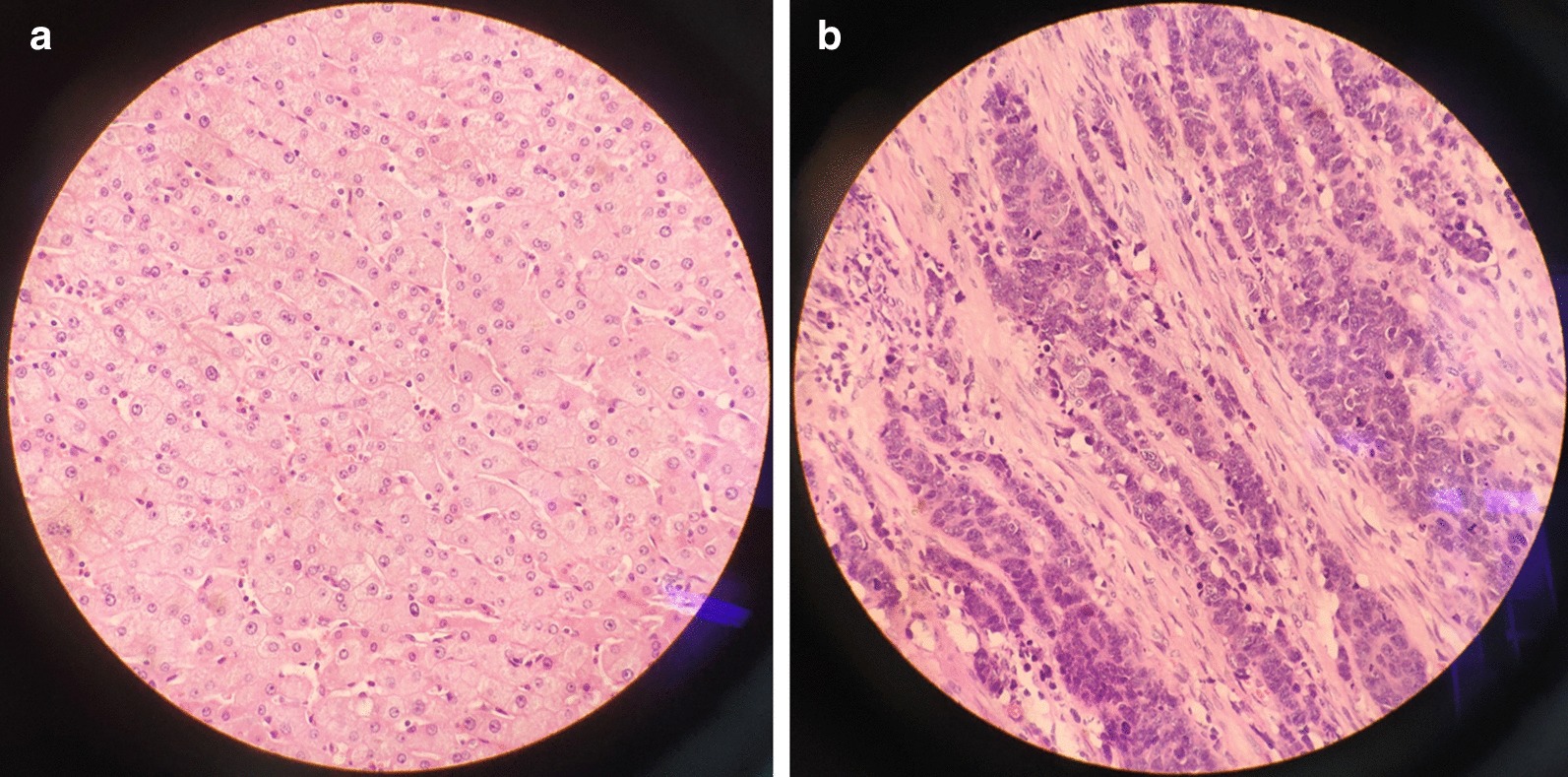
Postoperative histopathological examination of the resected specimen (hematoxylin and eosin stain, × 400). **a** Moderately differentiated hepatocellular carcinoma, **b** poorly differentiated gallbladder adenocarcinoma accompanied by neuroendocrine differentiation

Finally, postoperative histopathological examination (Fig. 5) proved synchronous primary double cancers in the liver and gallbladder that were moderately differentiated hepatocellular carcinoma and poorly differentiated gallbladder adenocarcinoma accompanied by neuroendocrine differentiation, with immunohistochemical markers AFP(−), CD19(+), CD56(+), and partly SYN(+). There was no microvessel invasion, no lymph node involvement and no perineural invasion.

The patient was discharged after 14 days and recovered well. The patient had an uneventful recovery over at least 24 months, without metastasis or recurrence. However, the patient was lost to follow-up, so her final outcome is unknown.

## Discussion and conclusions

The diagnostic criteria for MPC set by Warren and Cates are commonly accepted: (1) each tumor must present definite features of malignancy, (2) each tumor must be distinct, and (3) the chance of one being a metastasis of the other must be excluded [5]. Based on the different times that each primary cancer arises, they are divided into synchronous MPC and metachronous MPC, where synchronous MPC means a secondary malignancy occurring at the same time or within 6 months after the first malignancy [6]. Thus, we can conclude that the case presented can be regarded as multiple primary malignancies and synchronous double primary cancer. In MPC, liver cancer showed the fewest occurrences compared to other primary cancers [7], and synchronous HCC and GC is even more rare. Reviewing the previous literature, only 15 cases (Table 1) have been reported so far, with 6 in English [8–13] and 9 in Chinese. Among these, 11 cases were histologically proven as HCC and GC. Here, we present a case with the largest HCC and the poorest differentiation of GC. Furthermore, this is the first case reported in the literature that presented both HCC and GC accompanied by neuroendocrine differentiation.

**Table 1:** Cases of synchronous HCC and GC recorded in literature so far

Cases	Age(y)/sex	Size(cm) and histopathological feature		Prognosis/months
		Liver	Gallbladder	
Zhan-Guo Zhang [8]	65/F	6.2 × 4.5, CHC/NA	3 × 3, GC/w	8/died
Lu [9]	67/F	5.0 × 5.0, HNC/NA	NA, GC/NA	NA
Kim [10]	63/M	2.2 × 1.5, HCC/m	5 × 3, GC/m	17/alive
La Greca [11]		NA, HCC/w	Confined to the mucosa, GC/NA	NA
Taniai [12]	83/F	2.0 × 2.0, ICC/w	2.6 × 2.0, GC/NA	NA
Imada [13]	70/F	4.5 × 5.5, HCC/w	0.7 × 0.7, GC/w	4/died
In Chinese				
Xiao-xiao Yao	71/F	2.8 × 2.4, CHC/m	3.5 × 3.0, GC/m	10/died
He-bin Hou	56/F	5.0 × 3.0, HCC/m	1.9 × 1.5, GC/m	28/died
He-bin Hou	81/F	7.8 × 6.5, HCC/NA	Full of neoplasms in cavity, GC/NA	24/alive
Wei-lei Bao	78/F	5 × 5, HCC/w	1.0 × 1.0, GC/m	NA
Dan Qin	54/M	11.0 × 10.0, HCC/m	Invoved full layer, GC/m	2/metastasis
Wei-san Zhang	57/F	5.0 × 5.0, HCC/NA	Invoved full layer, GC/NA	3/died
Qing-wen Xu	49/F	8.0 × 6.0, HCC/NA	Invaded by HCC,GC/w	NA
Gang Wu	54/F	NA, HCC/w	NA, GC/NA	NA
Yi-ping Mou	67/F	4.0 × 2.0, HCC/NA	4.0 × 2.0, GC/w	NA

*M* male, *F* female, *CHC* combined hepatocellular cholangiocarcinoma , *NA* not available, *GC* gallbladder adenocarcinoma, *HNC* hepatic neuroendocrine carcinoma, *HCC* hepatocellular carcinoma, *ICC* intrahepatic cholangiocellular carcinoma, *m* moderately differentiated, *w* well differentiated

*This case existed the third cancer of the common bile duct adenocarcinoma

The pathogenesis of MPC has not been exactly clarified, although the etiological factors can be roughly grouped into host-related factors, lifestyle factors and environmental influences [14] as well as genetic factors and treatment-related factors [15]. Furthermore, it is commonly acknowledged that liver cancers are linked to alcohol consumption, virus infection or aflatoxin, while gallbladder carcinoma is linked to gallstones or inflammation. However, in this case, apart from gallstones, the patient appeared to have no other risk factors for MPC.

The preoperative diagnosis of MPC can be difficult, and gallbladder cancer with hepatic metastasis or liver cancer with gallbladder invasion can be mistakenly diagnosed. In this case, except for weakness and inappetence, no other discomfort was observed. A sharp rise in the serum level of AFP is usually linked with liver cancer, while CEA, CA19-9 and CA125 are linked with gallbladder adenocarcinoma, but these levels were normal in this patient. The giant tumor in the right lobe of the liver was noticed first so the tiny lesions in the gallbladder wall were nearly neglected. Six weeks after TACE was performed, an enhanced CT scan clearly demonstrated nodular thickness of the gallbladder wall up to 0.5 cm. To some extent, unobvious symptoms, insensitive serum examinations and atypical radiological manifestations made the diagnosis delayed and vague. Even though these assessments may mislead us to consider the possibility a primary HCC with satellite nodules in the gallbladder may exist, we finally denied this possibility because the two specimens showed totally different histopathological morphologies; moreover, the immunohistochemical markers revealed AFP(+) in liver specimens and AFP(−) in gallbladder specimens.

For all types of cancer, it is necessary to obtain as specific of a diagnosis as early as possible because different kinds of malignancies require various therapeutic methods. Few studies in the literature evaluate the different aspects that MPC involve and are also inconsistent [16], yet it is commonly agreed upon that the multiplicity of primary malignancies itself does not necessarily indicate a poor prognosis as long as an adequate diagnosis and treatment are performed [10]; overall survival depends on the most malignant tumor. According to the stage of each tumor, the pathological type and the patients’ physical condition, therapeutic strategies can be made [17]. When evaluating one primary malignancy, the possibility of finding another primary tumor must always be considered [18]. Radical surgery combined with postoperative chemotherapy or radiotherapy can achieve a longer survival time than conservative treatment [19]. However, metastatic cancers usually require palliative treatment.

In conclusion, synchronous primary double cancers in the liver and gallbladder display are rare and lack apparent radiological features. More precise and advanced diagnostic techniques are needed to obtain a clear diagnosis and refine treatment strategies. The management strategy should always be curative, even in the presence of multiple malignancies.

## Data Availability

The data that support the findings of this study are available from the corresponding author upon reasonable request. tjshibaomin@tongji.edu.cn.

## Abbreviations

MPC: Multiple primary cancers
HCC: Hepatocellular carcinoma
GC: Gallbladder adenocarcinoma
CT: Computed tomography
MRI: Magnetic resonance imaging
ALT: Alanine aminotransferase
AST: Aspartate aminotransferase
T-Bil: Total bilirubin
CEA: Carcinoembryonic antigen
CA19-9: Carbohydrate antigen19-9
CA12-5: Carbohydrate antigen 12-5
AFP: Alpha-fetoprotein
TACE: Transhepatic arterial chemoembolization
CD: Cluster differentiation
SYN: Synaptophysin
